# Large Bowel Obstruction in the Setting of Small Lymphocytic Lymphoma

**DOI:** 10.7759/cureus.9640

**Published:** 2020-08-10

**Authors:** Abigail W Cheng, Mahmuod Abdeljaber, Neiberg A Lima, Saad Shebrain

**Affiliations:** 1 Surgery, Western Michigan University Homer Stryker M.D. School of Medicine, Kalamazoo, USA; 2 Internal Medicine, Western Michigan University Homer Stryker M.D. School of Medicine, Kalamazoo, USA

**Keywords:** chronic lymphocytic leukemia, small lymphocytic lymphoma, large bowel obstruction

## Abstract

Large bowel obstruction (LBO) is a potential surgical emergency, commonly caused by colorectal carcinoma, diverticular stricture, and volvulus. LBO secondary to chronic lymphocytic leukemia (CLL) and small lymphocytic lymphoma (SLL) is a rare occurrence. We report an 81-year-old man with a history of CLL/SLL who presented to the emergency department with episodes of abdominal cramps and discomfort, diarrhea, vomiting, subjective flushes, and sweats. After a thorough evaluation, the patient was found to have a malignancy-mediated mechanical LBO at the hepatic flexure due to colonic compression by extensive pericolic lymphadenopathy. After resuscitation and medical optimization, an urgent laparotomy with oncologic right hemicolectomy was performed. Analysis of resected specimens, including lymph nodes, revealed atypical CD23- CLL/SLL cells. Postoperatively, aside from temporary ileus, the patient recovered well and was discharged home.

## Introduction

Chronic lymphocytic leukemia/small lymphocytic lymphoma (CLL/SLL) is a common malignancy of bone marrow origin in US adults, with a recent report projecting 21,040 new cases in 2020 [1]. CLL and SLL are both manifestations of the same disease, with the term CLL referring to the leukemic (hematological) phase of the disease where CLL cancer cells are found mostly in the blood and bone marrow, and the term SLL reserved for lymph node involvement [2].

Gastrointestinal (GI) involvement in CLL/SLL is uncommonly reported, ranging from 5.7% to 13% [3]. Additionally, large bowel involvement in CLL/SLL, including large bowel obstruction (LBO) secondary to lymph node compression specifically, is not commonly reported [4-6]. The two most serious complications of GI lymphoma include bowel perforation and obstruction [7,8]. Bowel perforation occurs in approximately 10% of cases and can lead to systemic sepsis and multiorgan failure [9]. Additionally, bowel obstruction can rapidly progress to a surgical emergency, depending on the location and disease progression. Therefore, rapid identification and prompt treatment of this disease are imperative to avoid adverse outcomes.

## Case presentation

An 81-year-old, tobacco-smoking Caucasian man with a history of CLL/SLL diagnosed 13 years ago, coronary artery bypass, and multiple abdominal wall hernia repairs presented to the emergency department (ED) with several hours of progressive abdominal discomfort, distension, diarrhea, sweats, and subjective flushes. He also had some belching and vomiting, but no hematemesis or hematochezia. While the patient had been experiencing subjective flushes since starting on ibrutinib (selective tyrosine kinase inhibitor), a potential side effect, for CLL/SLL one year ago, the flushes experienced on presentation were “very intense.” His colonoscopy three years ago revealed two benign polyps, which were removed, but no other abnormalities. On physical exam, the patient had dry mucous membranes but was normothermic with stable vital signs, except an elevated blood pressure of 150/92 mmHg. The abdominal exam showed a well-healed midline scar, distension, tympany to percussion, with a reducible incisional hernia on palpation. No tenderness to palpation or peritoneal signs were noted.

**Figure 1:**
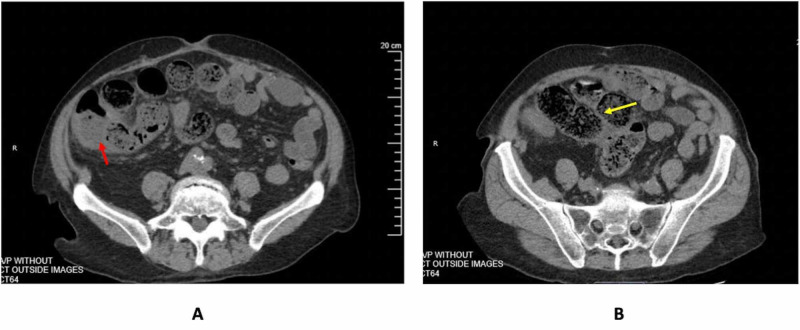
Abdomen/pelvis CT showing right colon thickening and edema (red arrow, A) and small bowel dilation with fecalization (yellow arrow, B).

**Figure 2:**
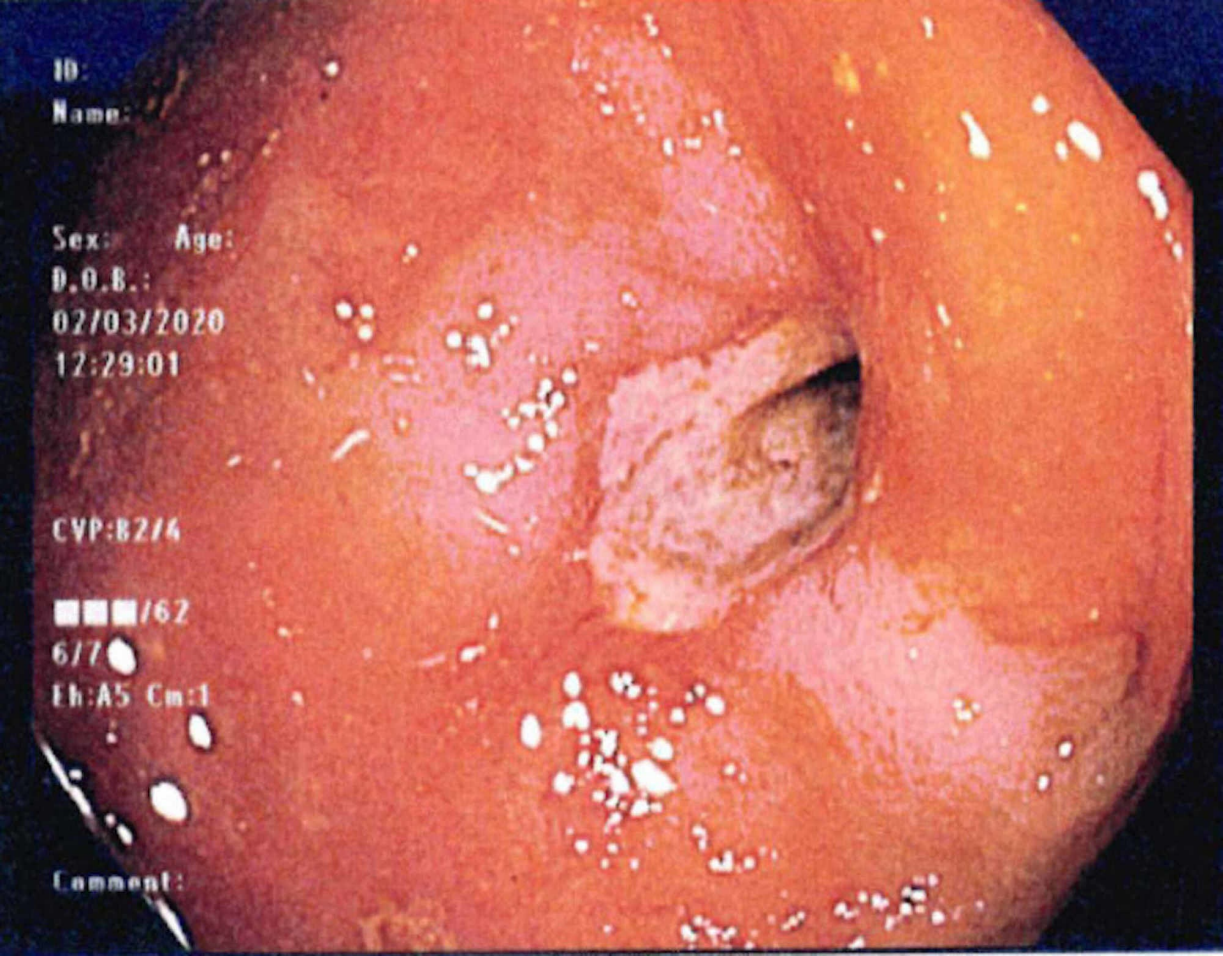
Colonoscopic view demonstrating a 90% obstructed lumen in the hepatic flexure of the colon.

On admission, laboratory studies were significant for moderate leukocytosis at 19.2 K/μL (normal range [NR] 4.5-11 K/μL) with elevated neutrophil count at 12.2 K/μL (NR 1.8-7.7 K/μL). Complete metabolic profile was within normal limits, aside from mild hypomagnesemia. Abdominal radiography showed small bowel and right colon dilation. CT scan demonstrated right colon thickening that was concerning for colitis versus mass, and small bowel dilation with possible fecalization, raising concern for disease in the terminal ileum/cecal area (Figure 1). After fluid resuscitation and correction of his hypomagnesemia, the patient underwent a diagnostic colonoscopy, which demonstrated at least 90% obstructed lumen in the hepatic flexure of the colon, highly concerning for malignancy (Figure 2). However, pathology from the colonoscopic biopsy showed non-neoplastic colorectal type mucosa and benign ulcers. Despite these findings and his history of benign colonoscopy, concern for malignant etiology was still high, given his history of CLL/SLL and tobacco use. Therefore, abdominal exploration was warranted according to his clinical status and lack of objective diagnosis.

Preoperatively, medical optimization was ensured, including gentle bowel preparation, administration of intravenous perioperative antibiotics, and venous thromboembolism prophylaxis. After patient consent for surgery was obtained, he underwent an uneventful exploratory laparotomy. Intraoperatively, the level of obstruction was found at the ascending colon secondary to colonic stricture and extrinsic compression by extensive lymphadenopathy. A right hemicolectomy, lymphadenectomy, and ileocolonic anastomosis were performed.

Postoperatively, the patient recovered slowly, aside from temporary postoperative paralytic ileus. This was not an unexpected complication, and improved gradually with conservative management. His electrolytes and blood panel were within normal ranges. He was discharged home on postoperative day 9 (12 days after admission) with the resolution of his abdominal symptoms, and continues to do well. He was restarted on ibrutinib for his CLL/SLL upon discharge.

**Figure 3:**
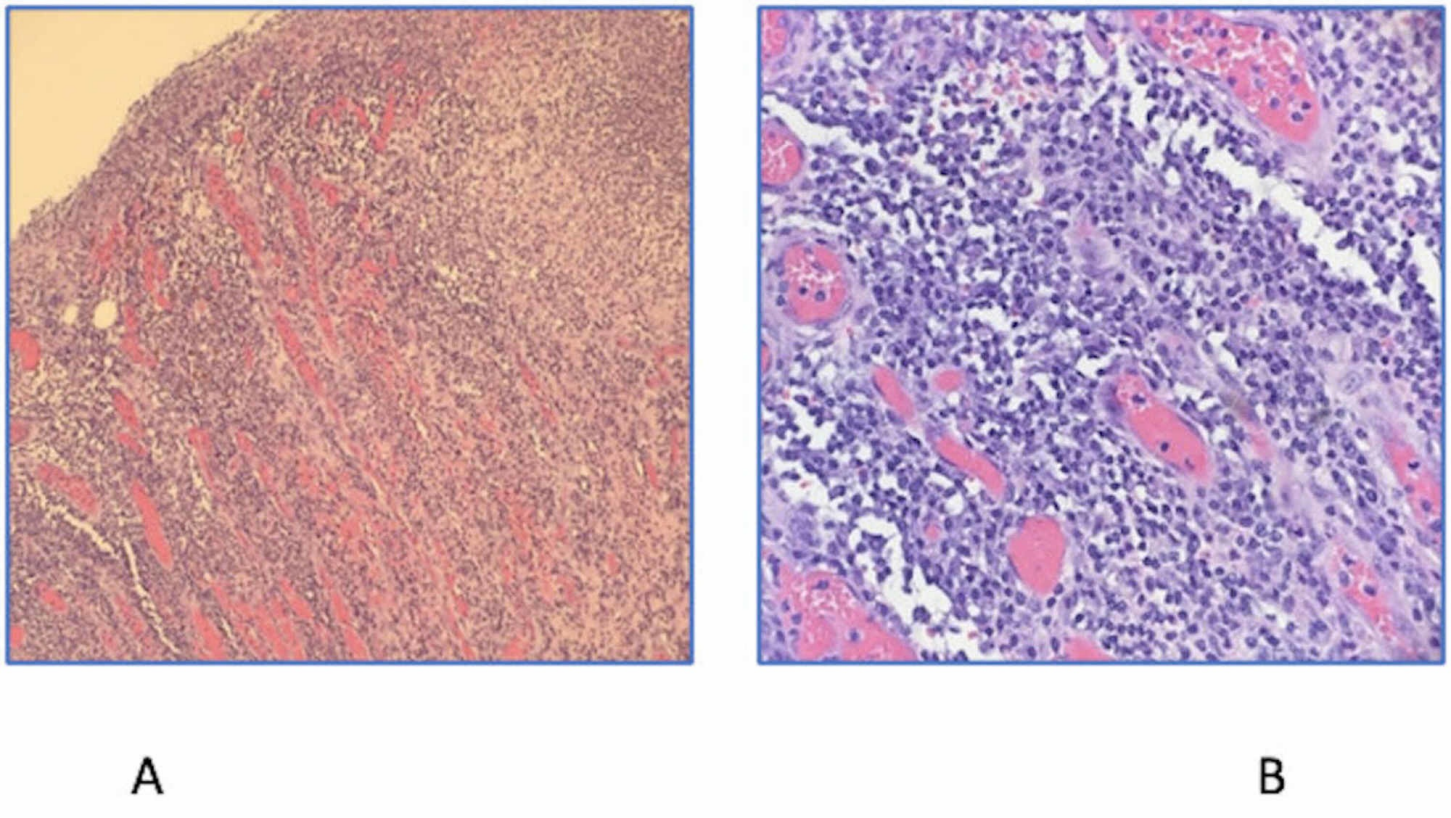
Colonic ulcer comprises fibrinopurulent exudate at the surface and underlying granulation tissue with chronic inflammatory cells and numerous plasma cells. Magnification of ×100 (A) and ×400 (B).

The final pathology confirmed the diagnosis. Gross pathology demonstrated normal terminal ileum, non-specific mucosal ulceration of the right colon, and extensive pericolic lymphadenopathy, with lymph nodes ranging from 0.3 to 2.4 cm in size. Microscopically, colonic ulcer demonstrated normal non-neoplastic colonic mucosa with some granulation tissue at the base of the ulcer with focal surface exudate (Figure 3). 

**Figure 4:**
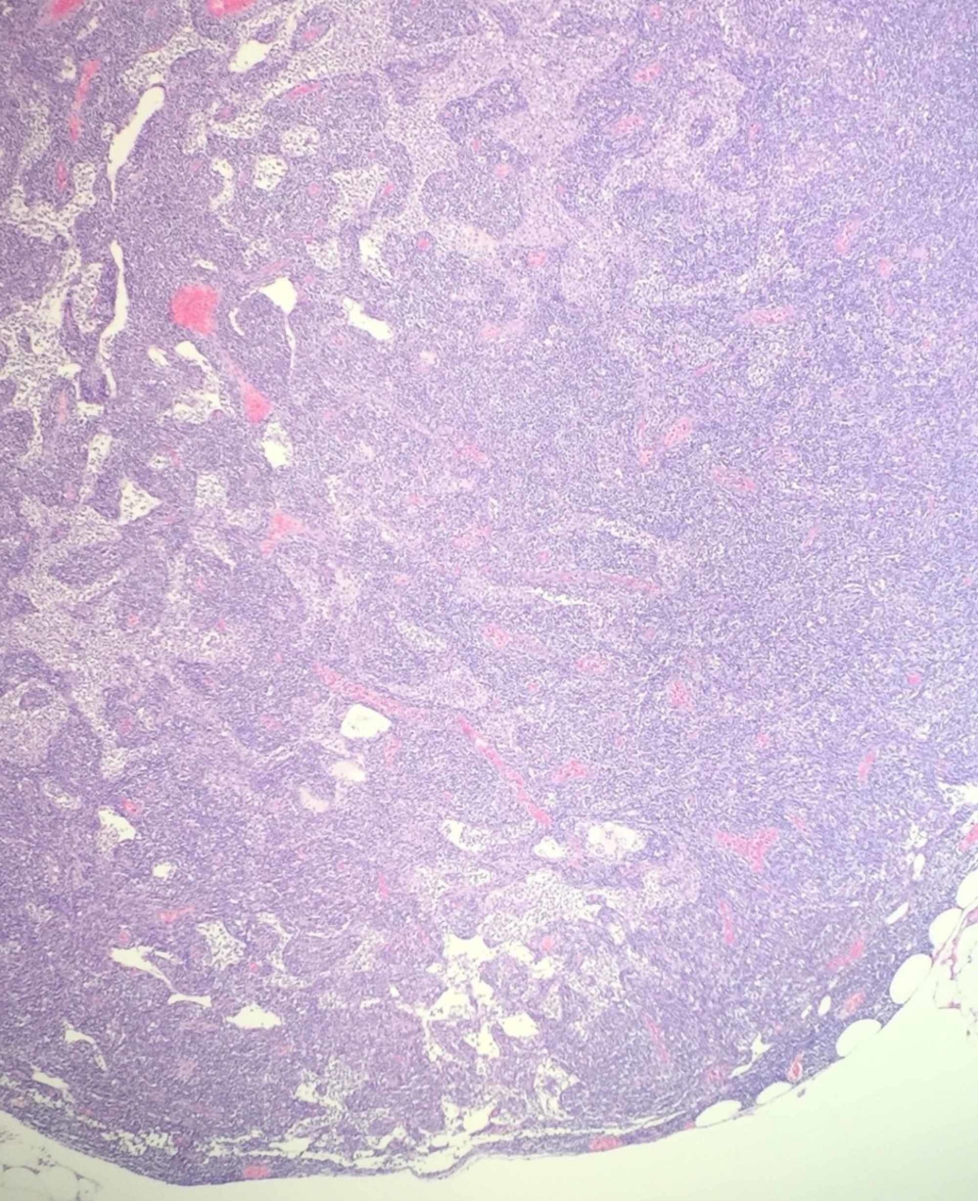
Pericolic lymph node with effaced architecture (no follicles with germinal centers are present), hematoxylin and eosin stain. Magnification of ×100.

Microscopic and flow cytometric analysis showed that more than 45 pericolic lymph nodes were involved by low-grade B-cell CD5+ lymphoma. Morphological features described effaced architecture by diffuse proliferation of small- to medium-sized lymphocytes with slightly irregular nuclear contours and mild plasmacytoid appearance (Figure 4). 

**Figure 5:**
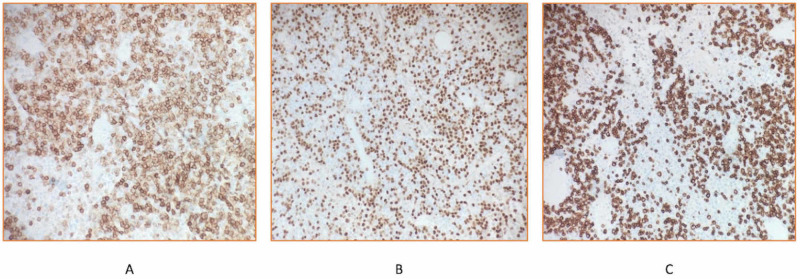
Immunohistochemical staining: CD5 membranous stain highlights atypical B lymphocytes (A) that also stains positive with PAX5 (nuclear stain, B) and negative for CD3 (CD3 highlights background T-cells) (C). All images have magnification of ×200.

**Figure 6:**
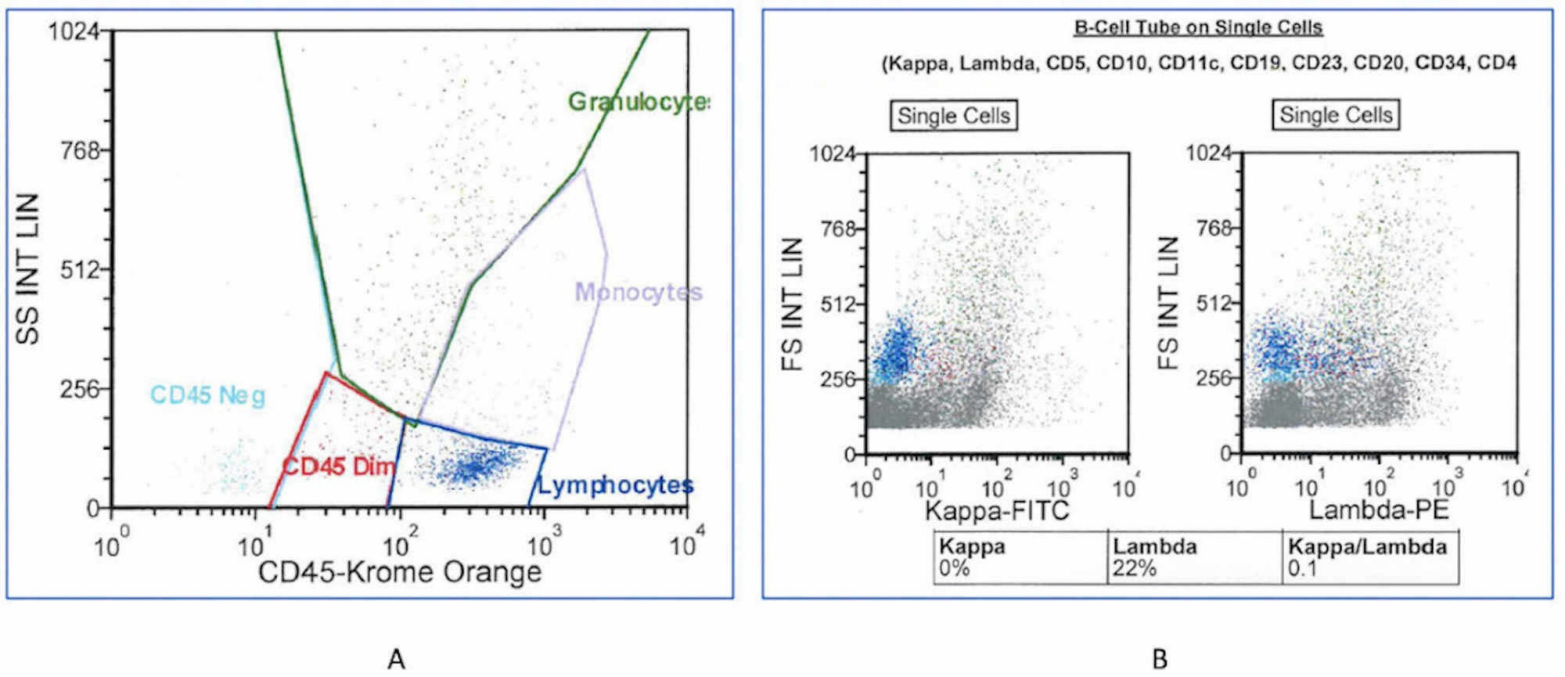
Flow cytometry characterizing cells of the pericolic lymph nodes (A) and their kappa and lambda ratio (B).

Immunohistochemical staining showed PAX5+ and CD20+ atypical B lymphocytes with CD5 co-expression, and negative for CD3 (Figure 5). Flow cytometry analysis further indicated monoclonal B cells that were negative for CD23, CD43, and cyclin D1. In addition, flow cytometry confirmed monoclonal origin (kappa/lambda ratio 0.1 [NR 0.26-1.65]) (Figure 6). 

## Discussion

Although the majority (~75%) of bowel obstructions occur in the small bowel, the remaining (25%) are caused by LBO, which still constitutes 2%-4% of all admissions requiring surgical intervention [10,11]. The three most common causes of LBO are colorectal cancer, diverticular stricture, and volvulus [12]. Clinically, most patients with LBO present with progressive abdominal distention, nausea, vomiting, crampy abdominal pain, and sometimes diarrhea, especially in cases of partial obstruction, such as in the case described. In addition to clinical examination, adjunct plain abdominal radiographs are essential in delineating the level of obstruction and are considered the first line in the workup, with a sensitivity and specificity of 84% and 72%, respectively [12,13]. However, CT of the abdomen and pelvis often remains the standard imaging modality to ascertain the potential cause and level of the LBO, where colon dilation is generally seen proximal to the obstruction [14]. While invasive, preoperative colonoscopy remains an essential diagnostic procedure in selected patients presenting with partial LBO, through which a biopsy of suspicious lesions can be obtained to confirm the diagnosis. It is prudent to use colonoscopies cautiously, as they can result in a closed-loop obstruction in patients with complete LBO, and increase the risk of bowel perforation. Patients with partial LBOs who are clinically stable could undergo a colonoscopy for therapeutic purposes, such as decompression. Additionally, endoscopic colonic stenting could be performed for patients who are poor surgical candidates as a temporary or permanent option.

Our case demonstrates a serious complication of longstanding CLL/SLL, presenting as an LBO secondary to pericolic lymphadenopathy. Large intestinal involvement in CLL/SLL is rare and is often reported as an extranodal infiltration of the mucosa/submucosa rather than involvement of lymph nodes [15]. There is a paucity of literature reporting LBOs caused by pericolic lymph node compression secondary to CLL/SLL. 

As LBO is not commonly reported in CLL/SLL, guidance for managing this condition specifically requires using principles related to the management of an LBO. Anatomic colon resection with or without anastomosis (colostomy) remains the standard approach in patients presenting with abdominal pain secondary to mechanical LBO [16,17]. In our patient, a right hemicolectomy with ileocolonic anastomosis was performed due to the location of the obstruction in the ascending colon.

It is important to confirm that CLL/SLL is the cause of a malignancy, especially if it involves the lymph nodes. Analysis of lymph node effacement and identification of cell markers and any translocations are imperative for the classification of CLL/SLL versus other lymphoma subtypes [2]. Mantle cell lymphoma is a competing diagnosis, defined by the t(11;14) translocation [2]. Our case had an interesting CD23- phenotype, as malignant cells in CLL/SLL are most commonly CD5+, CD20+, and CD23+; therefore, the lack of CD23 in our patient’s cells denotes an atypical presentation [18].

Finally, classifying new symptoms in patients with a history of CLL/SLL necessitates ruling out Richter syndrome, as GI involvement in CLL/SLL is generally reported after transformation to a diffuse large B-cell lymphoma (DLBCL) [6]. Richter syndrome often presents with acute onset B-symptoms, manifesting as fever, night sweats, and weight loss [19]. Despite our patient presenting with subjective flushes and sweats, these symptoms were more likely due to bowel obstruction. Additionally, microscopic examination of the patient’s lymph nodes revealed architectural effacement involving small, low-grade lymphocytes expanding in interfollicular regions of the lymph nodes, rather than large B-cells that are typically found in DLBCL [20]. These findings, in conjunction with flow cytometry confirming a monoclonal origin, are congruent with LBO secondary to CLL/SLL.

## Conclusions

Although rare, CLL/SLL should be considered in the differential diagnosis when evaluating a patient with LBO, especially if the patient has a history of CLL/SLL. Initial laboratory tests, including complete blood count, complete metabolic panel, and radiographic imaging, can provide guidance for the initial workup and severity of the obstruction. Once the patient is stabilized and causes for emergent operation are ruled out, a colonoscopy may be warranted to visualize large bowel pathology. Anatomic colon resection is an important and standard procedure for patients presenting with LBO. In the setting of CLL/SLL, a thorough pathologic evaluation of the resected specimens and lymph nodes is crucial for further management. A multidisciplinary team approach, in which surgeons and medical oncology teams are involved, provides the best evidence-based practice to achieve the best outcome for the patient. This approach ensures selection of the optimal treatment method for the LBO, and adequate follow-up on medication type and dosing for continued treatment of CLL/SLL.
